# 95. Prolonged respiratory viral infection associated with presence of coinfections in an urban birth cohort

**DOI:** 10.1093/ofid/ofac492.020

**Published:** 2022-12-15

**Authors:** Zheyi Teoh, Shannon C Conrey, Allison R Cline, Claire Mattison, Daniel C Payne, Monica McNeal, Rachel M Burke, Meredith L McMorrow, Ardythe L Morrow, Mary A Staat

**Affiliations:** Cincinnati Children's Hospital Medical Center, Cincinnati, Ohio; University of Cincinnati College of Medicine, Cincinnati, Ohio; University of Cincinnati College of Medicine, Cincinnati, Ohio; Centers for Disease Control, Atlanta, Georgia; US Centers for Disease Control and Prevention, Atlanta, Georgia; Cincinnati Children's Hospital Medical Center, Cincinnati, Ohio; CDC, Atlanta, Georgia; CDC/NCIRD/DVD, Atlanta, Georgia; University of Cincinnati College of Medicine, Cincinnati, Ohio; CCHMC, Cincinnati, Ohio

## Abstract

**Background:**

Prolonged infection by respiratory viruses has been reported, especially in hospitalized or immunocompromised children. However, little is known of factors contributing to prolonged respiratory viral infection, particularly in asymptomatic and less severe infections. We examined characteristics associated with prolonged viral infection in a community-based birth cohort.

**Methods:**

The PREVAIL cohort is a CDC-sponsored two-year birth cohort in Cincinnati, Ohio conducted during 4/2017 to 8/2020. Mid-turbinate nasal swabs were collected weekly from children and tested using the Luminex Respiratory Pathogen Panel. The primary outcome was prolonged viral infection, which was defined as a viral nucleic acid detection lasting 4 or more weeks. Proportions of prolonged viral infections were compared using Fisher’s exact test with Holms corrections. Adjusted odds ratios (aOR) and 95% confidence intervals were calculated using a mixed effects logistic regression model while controlling for within-subject clustering, viral species, child age, child sex, symptom status, and coinfection. This analysis was limited to subjects who provided at least 70% of weekly samples.

**Results:**

Among 101 children, providing 7871 child-weeks of follow-up, we identified 780 viral infections. The median duration of infection across all respiratory viruses was 1 week, except for bocavirus and coronavirus NL63, each with 2 weeks; 40% of bocavirus and >10% of adenovirus, coronavirus NL63, RSV A, human metapneumovirus, and parainfluenza 1 infections were associated with prolonged infection (>4 weeks). No prolonged infections were detected for influenza A or B, coronavirus 229E or HKU1, or parainfluenza 2 or 4 infections. Viral coinfection (aOR=3.1, 95% CI 1.9, 5.0) and female sex (aOR 1.8, 95%CI 1.1, 2.9) were significantly associated with prolonged infection, while symptom status and child age were not.

**Conclusion:**

In the PREVAIL cohort, detection of respiratory viruses lasting 4 weeks or longer was common for certain respiratory pathogens and was especially prolonged for bocavirus. Biological factors such as the presence of additional viral infections or child sex may affect the likelihood of prolonged infection.

**Disclosures:**

**All Authors**: No reported disclosures.

